# Tumor subtypes and signature model construction based on chromatin regulators for better prediction of prognosis in uveal melanoma

**DOI:** 10.3389/pore.2023.1610980

**Published:** 2023-06-09

**Authors:** Yue Li, Chao Xiong, Li Li Wu, Bo Yuan Zhang, Sha Wu, Yu Fen Chen, Qi Hua Xu, Hong Fei Liao

**Affiliations:** ^1^ School of Ophthalmology and Optometry, Nanchang University, Nanchang, Jiangxi, China; ^2^ Affiliated Eye Hospital of Nanchang University, Nanchang, Jiangxi, China; ^3^ National Clinical Research Center for Ocular Diseases Jiangxi Province Division, Nanchang, Jiangxi, China; ^4^ Jiangxi Clinical Research Center for Ophthalmic Disease, Nanchang, Jiangxi, China

**Keywords:** prognosis, uveal melanoma, TCGA, chromatin regulators, tumor subtypes

## Abstract

**Background:** Uveal Melanoma (UM) is the most prevalent primary intraocular malignancy in adults. This study assessed the importance of chromatin regulators (CRs) in UM and developed a model to predict UM prognosis.

**Methods:** Gene expression data and clinical information for UM were obtained from public databases. Samples were typed according to the gene expression of CRs associated with UM prognosis. The prognostic key genes were further screened by the protein interaction network, and the risk model was to predict UM prognosis using the least absolute shrinkage and selection operator (LASSO) regression analysis and performed a test of the risk mode. In addition, we performed gene set variation analysis, tumor microenvironment, and tumor immune analysis between subtypes and risk groups to explore the mechanisms influencing the development of UM.

**Results:** We constructed a signature model consisting of three CRs (RUVBL1, SIRT3, and SMARCD3), which was shown to be accurate, and valid for predicting prognostic outcomes in UM. Higher immune cell infiltration in poor prognostic subtypes and risk groups. The Tumor immune analysis and Tumor Immune Dysfunction and Exclusion (TIDE) score provided a basis for clinical immunotherapy in UM.

**Conclusion:** The risk model has prognostic value for UM survival and provides new insights into the treatment of UM.

## Introduction

**Uveal melanoma (UM)** is the most prevalent primary intraocular malignancy in adults [1, 2]. Primary UM is usually well controlled by surgery or radiotherapy, but metastases still occur in more than half of UM patients [3, 4]. UM most often metastasizes to the liver, and has a median survival of less than 1 year after metastasis [5–7]. Over the years, there has been an evolution in prognostic assessment (metastatic risk) of UM, from clinical and histological features to the analysis of genetic mutations and chromosomal abnormalities. These included clinical and histological features (patient age, tumor size, ciliary body involvement, extraocular extension, and so on), the genetic mutations [G protein subunit alpha q (GNAQ), G protein subunit alpha 11 (GNA11), Splicing Factor 3b Subunit 1(SF3B1), BRCA1 associated protein 1(BAP1), and Eukaryotic Translation Initiation Factor 1A X-Linked (EIF1AX)], and the composition of chromosomal anomalies of chromosome 3, 6 and 8 [8]. Some studies classify tumors and assess prognosis based on gene expression and chromosomal data [9, 10]. Despite extensive studies, the prognosis of UM has not significantly improved and there are no efficient therapies for metastatic UM, and treatments for metastatic UM such as immune checkpoint inhibitors (ICI), vaccination and t-cell therapy are much less effective than for other tumors [11–15]. Therefore, selecting key genomes for the stratification of UM patients and construction of tumor prediction models could provide new strategies for more precise molecular subtyping, screening of prognostic markers and potential therapeutic targets, and corresponding personalized treatment. At present, the rapid development of bioinformatics analysis is conducive to the screening of prognostic markers related to UM [16–18].

**Epigenetic alterations** can lead to aberrant gene regulation, which plays an important role in tumorigenesis by silencing tumor suppressor genes or activating oncogenes. These epigenetic changes include DNA methylation, histone modifications, and small non-coding RNA, many of which are associated with the initiation and progression of UM [19]. It has been found that mutations in BAP1, SF3B1, and EIF1AX in UM with different prognoses exhibit different types of methylation cluster status, and hypermethylation of chromosome 3 in UM is also associated with downregulation of BAP1 gene expression, which was further confirmed *in vitro* experiments that knockdown of BAP1 gene or deletion of the protein induces effects on methylation status in UM cells, causing UM cells to exhibit a low metastatic risk phenotype [20–22]. Histone modifications have also been associated with UM metastasis and proliferation [23–25]. MicroRNAs, the most widely studied small non-coding RNAs, have been shown to have dysregulated anti-apoptotic effects, accelerated cell cycle progression, and enhanced invasion and metastasis of many cancers [26]. Epigenetic mechanisms have been found to regulate the expression and activation of miRNAs in UM, which in turn regulate the progression of UM [27, 28]. These studies suggest that epigenetic alterations are closely associated with the onset and progression of UM and that epigenetic mechanisms play an important role in the development of UM.

**Chromatin regulators (CRs)** are drivers of epigenetic alterations and are classified by function as DNA methylators, histone modifiers, and chromatin remodelers [29–31]. In recent years, abnormal expression of CRs is closely associated with the development of several diseases, including several cancers [32–35]. Aberrant expression of CRs CHD8 and CTCF led to abnormal chromatin structure and epigenetic changes in many cancer-associated genes, ultimately leading to tumor progression and metastasis in prostate cancer patients [36]. Several CRs associated with cancer subtypes and prognosis have been identified as potential drivers of carcinogenesis [37]. There were very few studies on the role of CRs in UM, so a comprehensive analysis of CRs may make a theoretical contribution to the diagnosis, classification, prognostic assessment, and other features of UM. The simultaneous screening of key genes for the stratification of UM patients and the construction of tumor prediction models may provide new strategies for more precise molecular typing and corresponding personalized treatment.

In this study, we applied bioinformatics analysis to identify the key regulators and prognostic genes of CRs in UM. We applied the Non-negative Matrix Factorization (NMF) to cluster The Cancer Genome Atlas (TCGA) dataset based on the expression levels of prognostic-related CRs genes in UM, and the differences in patient prognosis and clinical traits between subtypes were also studied. We also screened core genes using protein interaction networks and constructed prognostic risk models using univariate Cox regression, least absolute shrinkage and selection operator (LASSO) regression, and multivariate Cox regression analysis. Then the accuracy, independence, and validity of the risk model were assessed in the training cohort and validation cohort. In addition, the relationship of the risk model with the tumor microenvironment, immune infiltration and immune checkpoints, and drug sensitivity was investigated, thereby expanding the risk model prognostic values for patients with UM. In summary, our work constructs a new risk model based on CRs gene expression levels associated with UM prognosis, which may have implications for the development of diagnosis and treatment of UM.

## Materials and methods

### Data download and collation

A list of CRs (870 genes) was collected from previous research [37], and the list of the CRs gene name was shown in Supplementary Table S1. The mRNA data and clinical and pathological characteristics were obtained from three datasets, including the TCGA-UM downloaded from the public dataset TCGA (The Cancer Genome Atlas, https://portal.gdc.cancer.gov/) and the UCSC Xena website (https://xena.ucsc.edu/), the GEO database (Gene Expression Omnibus, https://www.ncbi.nlm.nih.gov/geo/) datasets GSE22138and GSE84976 [38, 39]. All data were normalized using R software, and the ComBat method from the “SVA” R package was used to remove the batch effects among three datasets [40]. The Principal Component Analysis (PCA) showed the batch correction of three datasets.

### Classification validation and variance analysis of TCGA data sets

First, CRs genes significantly associated with UM prognosis were screened by univariate Cox analysis, and prognosis-related CRs genes were treated with the R package “survival” with selection *p* < 0.01. Unsupervised subgroups of TCGA-UM datasets were identified using the R package “NMF” [41], based on these prognosis-related CRs genes. The subtypes were verified by PCA using the R package “ggplot2.” The Kaplan–Meier survival curves of the different subgroups were analyzed and plotted using the R packages “survival” and “survminer” [including overall survival (OS) and progression-free survival (PFS)].

To explore the differences in clinical and pathological traits, tumor microenvironment, and tumor immunity between the two subgroups. The gene expression of prognosis-related CRs genes and clinical traits in different subtypes were visualized using the R package “Heatmap” and “ggplot2,” respectively. The KEGG pathway was analyzed to explore the differences in the biological processes between the different subgroups using the R package “GSVA.” The ESTIMATE algorithm calculated immune, stromal, and ESTIMATE scores in different subtypes by using the R package “estimate.” A ssGSEA algorithm was used to investigate the immune cell infiltration relationships between the different subgroups using the R package “GSVA” and “GSEABase,” R package “reshape2,” “ggpubr,” and “pheatmap” was used to draw a heatmap and differential boxplot.

### Screening for key genes

To further screen the key CRs genes, the protein regulatory networks of the prognosis-related CRs genes were analyzed using the STRING online database, and only interactions that enjoyed a minimum required combined score >0.4 were set as significant, and visualized the results by Cytoscape software. Furthermore, the key genes were identified based on the PPI network by using cytoHubba, which is another plug-in of Cytoscape. We defined the genes with node (degree > 9) as key genes and used them for subsequent study analysis.

### Risk model construction

Based on key CRs genes expression profiles and survival data were combined for further analysis, and to further minimize the dimensionality and build the risk signature, The R package “glmnet” and “survminer” were used to perform the least absolute shrinkage and selection operator (LASSO) regression analysis to determine the minimum lambda value of the lasso model, and further reduce the number of genes by multivariate Cox analysis to determine the final genes and coefficients that constitute the risk model. The patients’ risk scores were then determined. Before that, the TCGA dataset was randomly grouped to obtain the TCGA training group (*N* = 56) and testing group (*N* = 28). The risk score formula for the sample is as follows:

Risk score = (Coef1 *mRNA1 expression) + (Coef2 * mRNA2 expression) +…+ (Coef n *mRNA n expression).

The verification datasets GSE22138 and GSE84976 were categorized into high- and low-risk groups based on the risk score’s median value in the TCGA training group.

### Risk model validation

To assess the prognostic value of the risk model, we used the following approaches. First, the predictive ability of the risk model was assessed by using the R package “survminer” and “timeROC,” and the Kaplan-Meier curves, with *p* < 0.05 between the two groups indicating a significant difference in overall or progression-free survival. ROC curves at 1, 3, and 5 years were used to measure the accuracy of the predictive ability of the risk model, with AUC (Area Under ROC Curve) value > 0.7 as a valid criterion. Then, the risk model was compared with other clinical characteristics. Finally, the “rms” R package plotted the clinical nomogram. The performance of the nomogram in predicting the overall survival (OS) of UM patients was evaluated using factors such as sex, age, stage, TNM staging of the tumor, and risk score. The calibration curve then proved the nomogram’s efficacy.

### Gene set variation analysis

To explore the potential molecular mechanisms affecting prognosis, we performed functional enrichment analysis of genes in high- and low-risk groups. The KEGG pathway was analyzed to explore the differences in the biological processes between the high and low-risk group using the R packages “GSVA.”

### Tumor microenvironment and immune landscape analysis

To confirm whether the CRs characteristics of the risk model were correlated with the tumor microenvironment and tumor immunity, we assessed the differences in ESTIMATE scoring and immune cell infiltration between the two groups. The ESTIMATE algorithm calculated immune, stromal, and ESTIMATE scores in different subtypes by using the R package “estimate.” Correlation tests were used to calculate the correlation between the three signature genes and risk scores and the expression of immune checkpoint-associated genes. The TIDE algorithm was applied to predict the response to immunotherapy in both high and low-risk groups.

### Analysis of signature genes in the risk model

To observe the distribution of TCGA samples in terms of subtyping and risk grouping, we performed an analysis using R packages “ggalluvial” and “ggplot2,” and presented the results with a Sankey diagram. The distribution of 80 TCGA UM patients in subtypes and risk subgroups was statistically and consistently analyzed, and the results were presented in a 2 × 2 contingency table, while SPSS software was applied to calculate the kappa coefficient to verify the consistency of the two classifications. In addition, we compared whether risk scores differed between subtypes, and the expression and survival analysis of signature genes in risk models between risk subgroups was done by R packages “limma” and “survminer.” Dividing the dataset samples into high and low gene expression groups by the median value of characteristic gene expression (which varies across datasets).

### Statistics analysis

All statistical analyses were performed in R software (version 4.2.0). *p* < 0.05 was considered statistically significant unless otherwise stated. The consistency test was calculated by SPSS software, and a kappa coefficient (κ) greater than 0.61 indicated a good agreement [42, 43].

## Results

### Detailed clinical data from three study cohorts

A total of three cohorts were used in this study: 80 UM samples that came from TCGA-UM. The datasets GSE22138 with 63 samples and GSE84976 with 28 samples. The detailed baseline data, clinical and pathological characteristics, and survival time of the three cohorts were presented in Table 1. Three data sets were calibrated by combat functions, and PCA showed successful correction of batch effects (Supplementary Figure S1).

**TABLE 1:** The detailed baseline data and clinical characteristics of the three cohorts.

Characteristics	Training cohort	Validation cohort	
	(TCGA-UM, *n* = 80)	GSE22138 (N = 63)	GSE84976 (N = 28)
Age at diagnosis, years			
≤65	46	36	13
>65	34	27	15
Gender			
Female	35	24	—
Male	45	39	—
Stage			
Stage I	0	—	—
Stage II	36	—	—
Stage III	40	—	—
Stage IV	4	—	—
T			
T1	0	—	—
T2	5	—	—
T3	36	—	—
T4	39	—	—
N			
N0	76	—	—
Unknown	4	—	—
M			
M0	73	—	—
M1	4	—	—
Unknown	3	—	—
Tumor location			
Posterior to equator	67	54	—
Anterior to equator	5	3	—
All over the eye	8	1	—
Unknown	0	5	—
Tumor diameter			
≤15 mm	24	27	—
>15 mm	55	26	—
unknown	1	10	—
Tumor thickness			
≤10 mm	37	12	—
>10 mm	43	51	—
Extrascleral extension			
Yes	7	5	—
No	68	48	—
Unknown	5	10	—
Person neoplasm cancer status			
Tumor free	56	—	28
With tumor	9	—	0
Unknown	15	—	0
OS(years)	0.01∼7.12	—	1.17∼13
PFS(years)	0.01∼6.84	0.01∼10.05	—

### Typing and analysis of CRs-based subtypes

**FIGURE 1:**
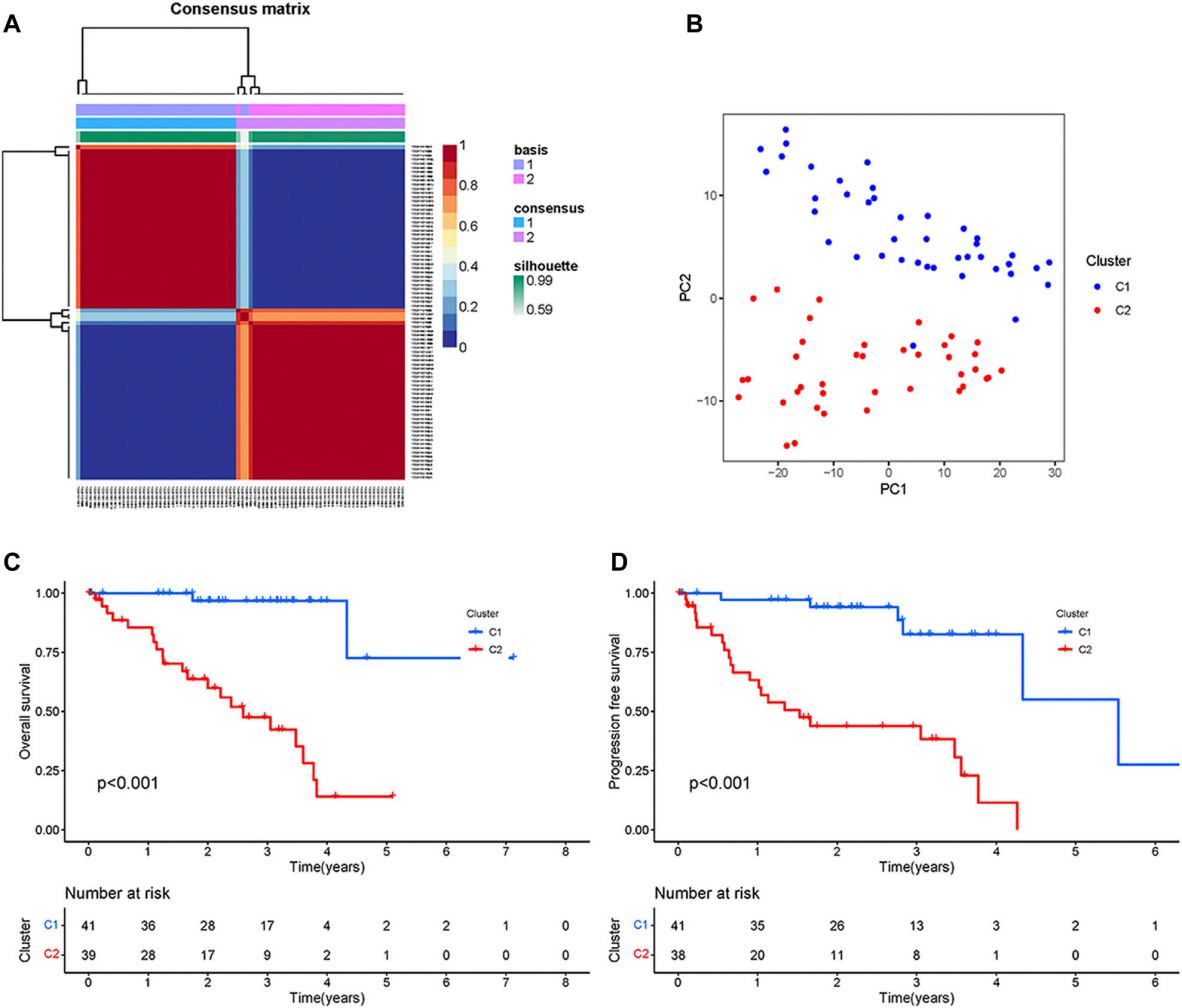
Typing and Identification of CRs-based Subtypes: **(A)** The cluster heatmap of tumor subtypes. **(B)** PCA showed a significant difference between the two subtypes. **(C)** Survival curves of overall survival for patients with two subtypes. **(D)** Survival curves of progression-free survival for patients with two subtypes.

First, the univariate Cox regression analysis identified 111 CRs genes that were significantly associated with UM prognosis, and the results of the analysis are shown in Supplementary Table S2. Then, based on the expression of these 111 genes, the TCGA samples were divided into two subtypes (namely C1, *n* = 41, and C2 *n* = 39) by NMF analysis according to the results of the heatmap of subtypes (Figure 1A). As shown by the results of PCA analysis (Figure 1B), 80 patients were well grouped into two distinct subtypes. The Kaplan-Meier (KM) curve results showed that the C2 subtype was the high-risk group with significantly shorter OS and PFS (*p* < 0.001) (Figures 1C, D). These results indicated that TCGA-UM can be classified based on prognosis-related CRs genes.

**FIGURE 2:**
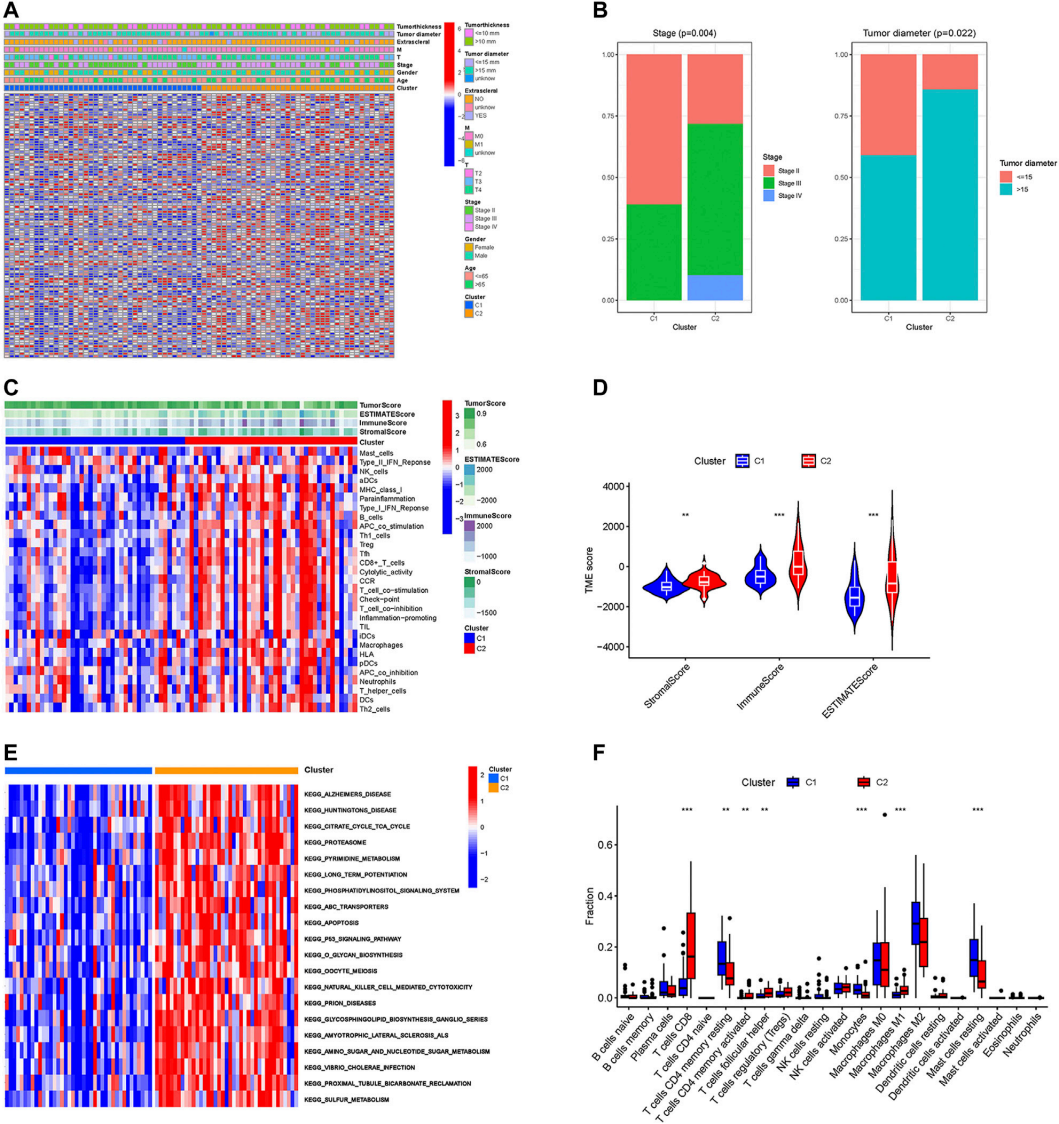
Different characteristics between two subtypes. **(A)** The heatmap of expression levels of prognosis-related CRs genes and clinical traits in the two subgroups. **(B)** Percentage distribution of clinical traits with significant differences between the two subtypes. **(C)** The ssGSEA analysis of the immune cell infiltration level in the two subgroups. **(D)** Difference analysis of TME scores. **(E)** KEGG pathway analyses of GSVA in the two subgroups. **(F)** Boxplot of the abundance of immune cells in the two subgroups. **p < 0.01, ***p < 0.001.

To further explore the differences between these two subgroups, we analyzed the distribution of prognosis-related CRs genes and clinical traits among different subtypes. As the results in Figure 2A showed that the expression of prognosis-related CRs genes showed a difference between the two subgroups, with differences in tumor stage and tumor diameter among clinical traits (*p* < 0.05) (Figure 2B). The results indicated that subgroup C2 had a distinct pattern of immune infiltration compared to subgroup C1 (Figure 2C), and the results of the analysis of tumor microenvironment and immune infiltration levels between the two subtypes are shown in Figure 2D, the stromal, immune, and estimate scores of the C2 subgroup were higher than those of the C1 subgroup, and the differences were statistically significant (*p* < 0.05). The results of the different KEGG pathways within the two subtypes showed that subgroup C1 was highly enriched in substance metabolism (including pyrimidine metabolism, amino sugar and nucleotide sugar metabolism, and sulfur metabolism), and in addition, the C2 subgroup was also highly enriched in apoptosis, P53 signaling pathway, and O-glycan biosynthesis (Figure 2E), all of which KEGG pathways are closely associated with tumors. Furthermore, subgroup C2 had a significantly higher abundance of immune cells—including CD8 T cells, activated memory CD4 T cells, follicular helper T cells, and Macrophages M1, while the abundance of immune cells (including resting memory CD4 T cells and resting Mast cells) was significantly higher in subgroup C1 than in subgroup C2 (Figure 2F). These results suggest that the two subgroups have different characteristics in terms of the KEGG pathway, tumor microenvironment, and immune infiltration level.

### Selection of key CRs genes

**FIGURE 3:**
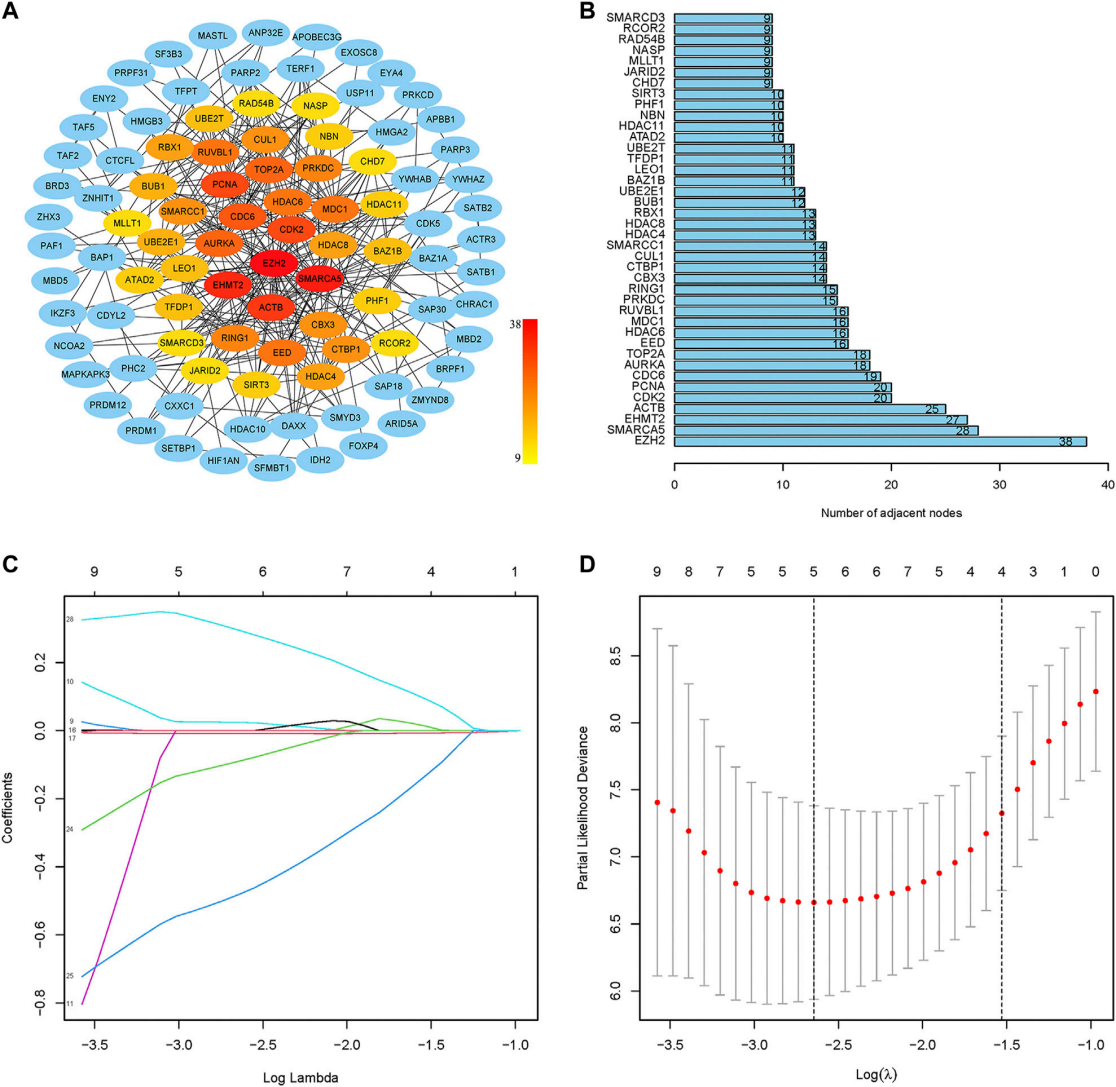
Screening of key CRs genes and construction of risk model. **(A)** The PPI network of prognostic CRs genes. **(B)** The names and number of nodes of the top 40 genes in the PPI network. **(C)** Coefficient curve. Different colors represent different genes. **(D)** The minimum lambda value of the lasso model, λ = 5, was determined at the minimum deviation from the partial likelihood, and five genes were available for the analysis.

The PPI network of prognostic-related CRs genes were shown in Figure 3A, with the help of the String database analysis and Cytoscape software, direct or indirect functional interactions of proteins were visualized, and core genes were specifically marked (the darker the red color, the higher the number of nodes). The names and number of nodes of the key genes are shown in Figure 3B, and these 40 genes were defined as key CRs genes for subsequent study analysis.

### Establishment and validation of CRs-based signature risk model

First, the random grouping of the TCGA training and test groups was reasonable, with no bias in the selection of clinical traits (*p* > 0.05). Statistical data of each group are shown in Table 2. Then, LASSO regression analysis successfully screened out 5 CRs including CDK2, CUL1, RUVBL1, SIRT3, SMARCD3, and multifactorial Cox analysis was performed on these 5 genes (Figures 3C, D), and finally, a risk model consisting of 3 genes (RUVBL1, SIRT3, and SMARCD3) were successfully constructed (Table 3).

**TABLE 2:** Statistical analysis of clinical features of a randomized grouping of TCGA dataset.

Covariates	Type	Training group	Testing group	Total group	*p*-value
Age	≤65	33 (58.93%)	13 (54.17%)	46 (57.5%)	0.8823
	>65	23 (41.07%)	11 (45.83%)	34 (42.5%)	
Gender	Female	23 (41.07%)	12 (50%)	35 (43.75%)	0.6229
	Male	33 (58.93%)	12 (50%)	45 (56.25%)	
Stage	Stage II	28 (50%)	8 (33.33%)	36 (45%)	0.0975
	Stage III	24 (42.86%)	16 (66.67%)	40 (50%)	
	Stage IV	4 (7.14%)	0 (0%)	4 (5%)	
M	M0	50 (89.29%)	23 (95.83%)	73 (91.25%)	0.4047
	M1	4 (7.14%)	0 (0%)	4 (5%)	
	Unknown	2 (3.57%)	1 (4.17%)	3 (3.75%)	
N	N0	53 (94.64%)	23 (95.83%)	76 (95%)	1
	Unknown	3 (5.36%)	1 (4.17%)	4 (5%)	
T	T2	5 (8.93%)	0 (0%)	5 (6.25%)	0.3081
	T3	25 (44.64%)	11 (45.83%)	36 (45%)	
	T4	26 (46.43%)	13 (54.17%)	39 (48.75%)	

**TABLE 3:** Genes in the prognostic signatures of the risk model.

Gene symbol	Full name	Risk coefficient
RUVBL1	RuvB-like AAA ATPase 1	−0.52462896083222
SIRT3	Sirt3, Silent Mating Type Information Regulation 2 Homolog 3	−1.12003130376948
SMARCD3	BAF60C/SWI-SNF related, matrix associated, actin dependent regulator of chromatin, subfamily d, member 3 protein	0.837694828472529

Risk score = (−0.52462896083222 × RUVBL1 expression) + (−1.12003130376948 × SIRT3 expression) + (0.837694828472529 × SMARCD3 expression).

The median risk score (1.324375242) of the TCGA training group was used as the threshold to distinguish the high-risk group from the low-risk group. Based on the grouping, a total of five cohorts were available for assessing and validating the prognostic value of the model in this study, including the TCGA training group (*N* = 56), the TCGA testing group (*N* = 24), the TCGA all group (*N* = 80) and two independent datasets GSE22138 (*N* = 63) and GSE84976 (*N* = 28).

### Validation of the risk model

**FIGURE 4:**
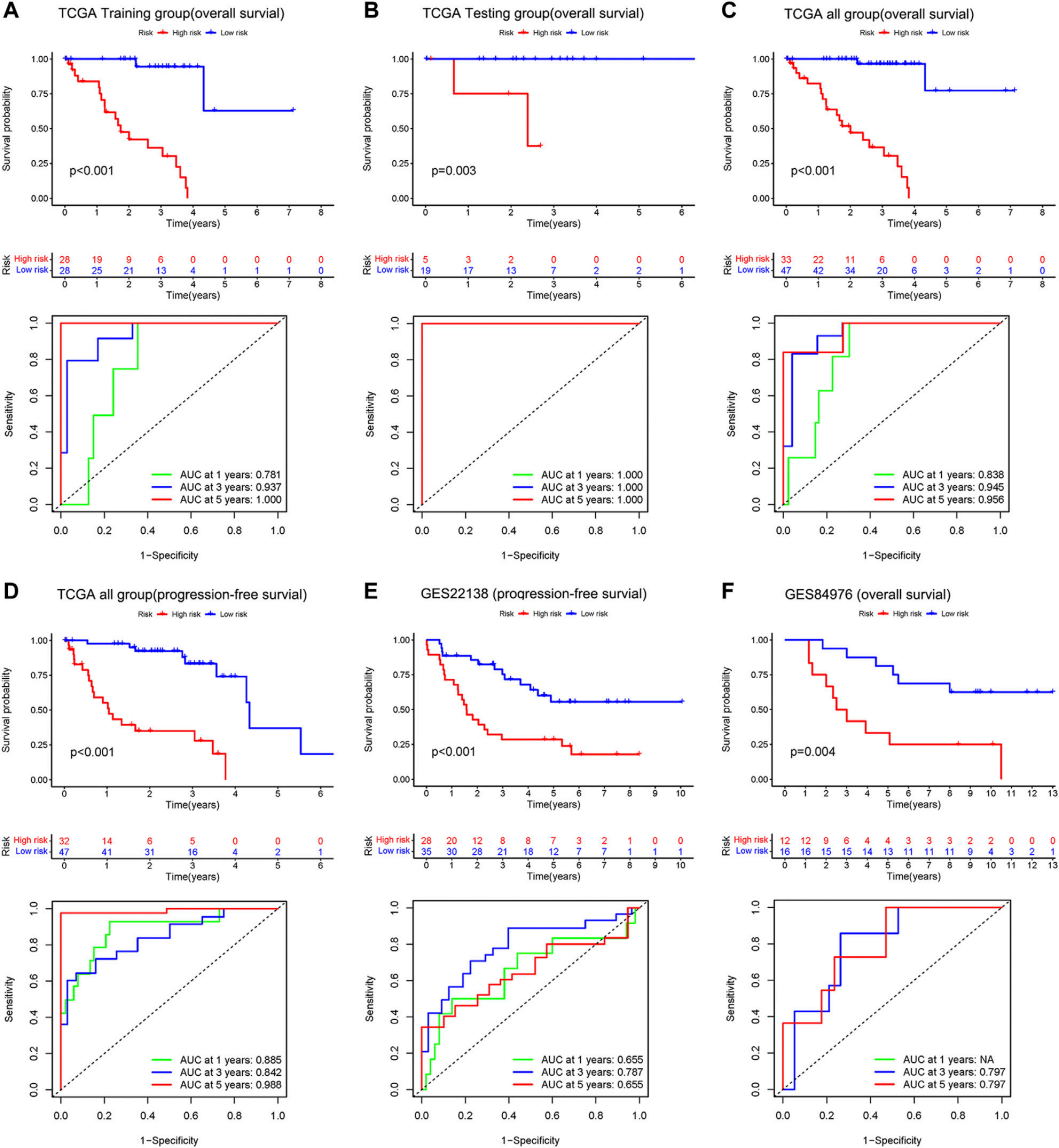
Upper: Kaplan–Meier survival analyses based on the risk model. Bottom: 1 -, 3-, and 5-year ROC analyses based on the risk model. Overall survival of patients in the TCGA training group **(A)**, the internal validation group of the TCGA testing group **(B)**, the entire TCGA dataset **(C)**, and the independent dataset GSE84976 **(F)**. Progression-free survival of patients in TCGA dataset **(D)** and the independent dataset GSE22138 **(E)**.

The results of Kaplan-Meier survival curves for all datasets are shown in Figures 4A–F, patients with a high-risk score tended to have a lower survival probability and die (or metastasis) earlier than those with a low-risk score. The AUC values for the rest of the dataset exceeded 0.7, except for the 1-year and 5-year AUC values of 0.655 for GSE22138. The results suggest that our risk model has a good predictive effect on the prognosis of UM patients.

**FIGURE 5:**
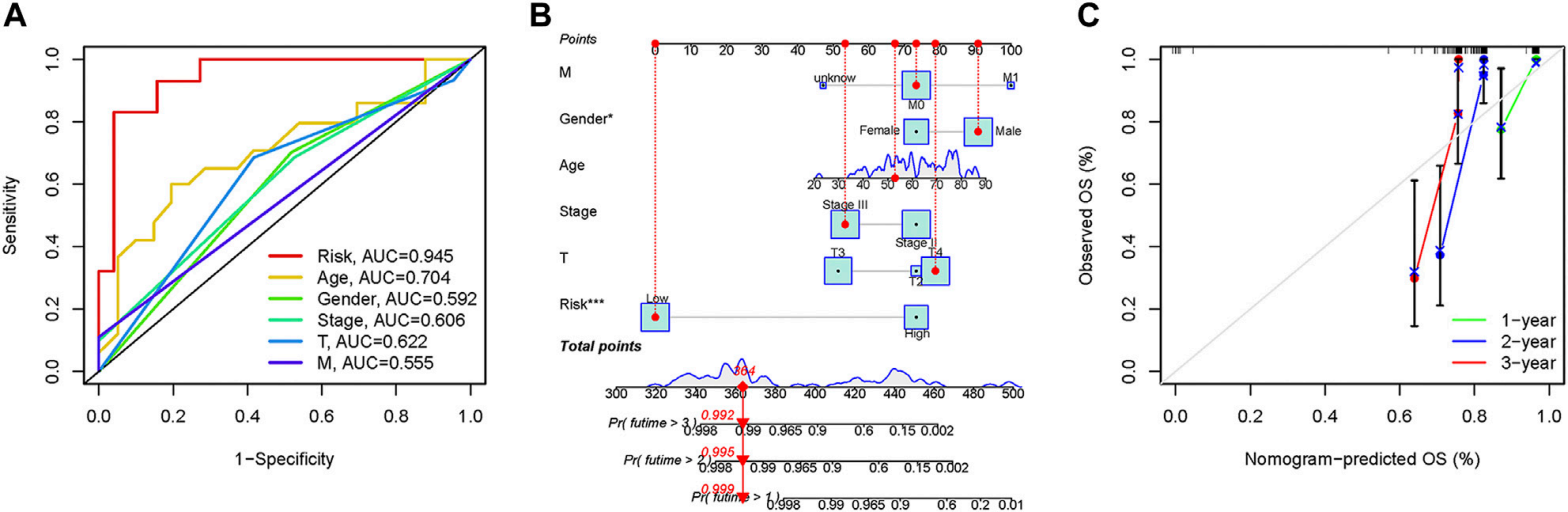
Prognostic assessment efficiency comparison of the ROC curve and construction of the nomogram based on risk score and clinical factors. **(A)** ROC curve analysis at 3 years. **(B)** The nomogram to predict the 1-, 2- and 3-year survival risk of UM patients. **(C)** Calibration curve for the 1-, 2-, and 3-year predicted survival nomogram. *p < 0.05, ***p < 0.001.

In the 3-year ROC curves, the AUC values for risk scores were greater than those for other clinical traits, suggesting that the use of risk scores predicted the survival of UM patients better than other clinical traits (Figure 5A). Furthermore, we developed a prognostic nomogram for estimating the UM patients’ survival likelihood (Figure 5B). This prognostic nomogram could systematically anticipate the 1-, 2-, and 3-year OS of UM patients. The calibration curve showed that actual results were consistent with predicted results (Figure 5C).

### Gene set variation analysis

**FIGURE 6:**
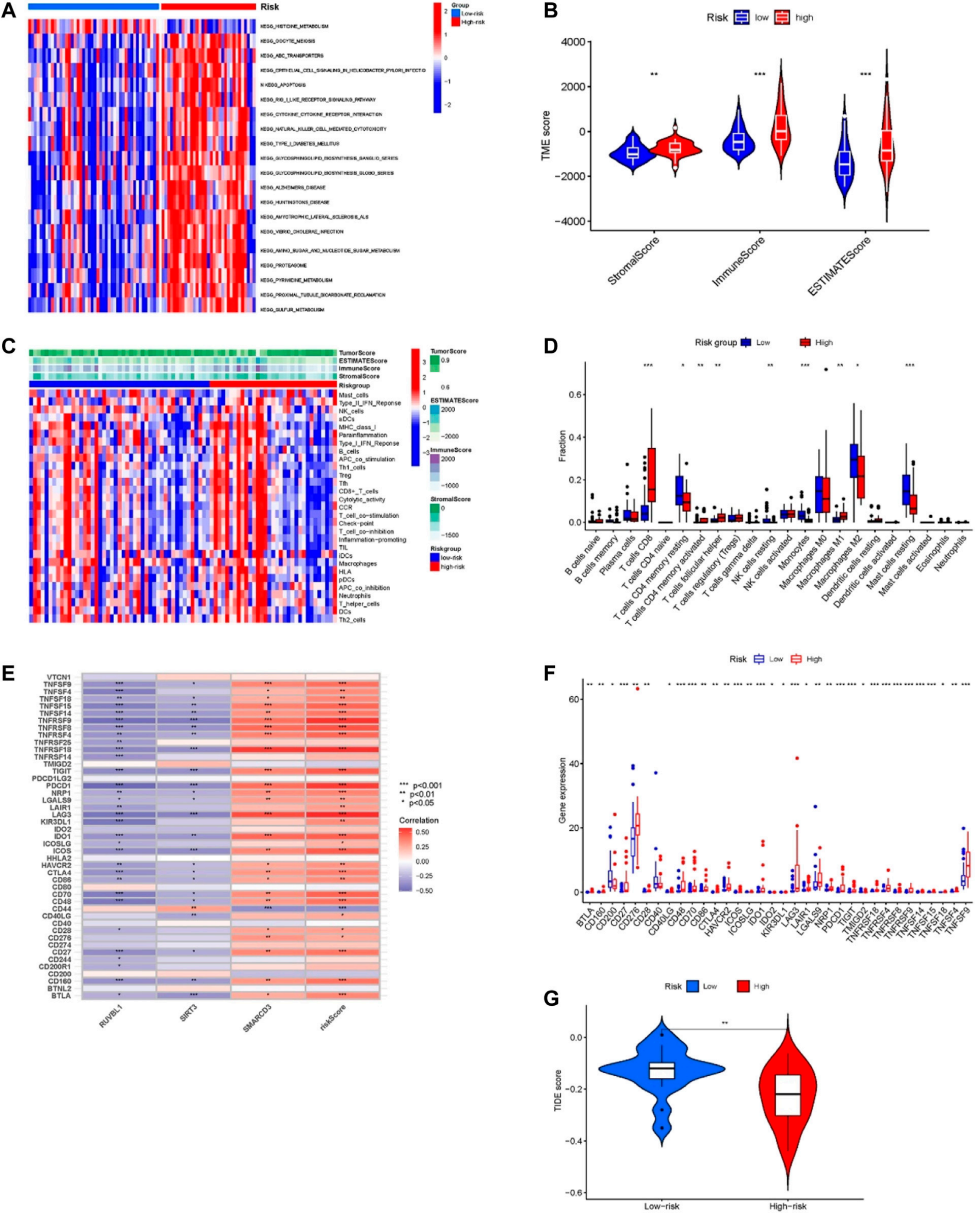
Tumor microenvironment and immune-related analysis in two groups. **(A)** KEGG pathway analyses of GSVA in the two groups. **(B)** Difference analysis of TME scores in two groups. **(C)** The ssGSEA analysis of the immune cell infiltration level in the two subgroups. **(D)** Comparison of immune cell infiltration in two groups. **(E)** Correlation of risk score and 3 risk model-related genes with the expression of immune checkpoints. **(F)** Comparison of the expression of immune checkpoints in two groups. **(G)** Difference analysis of TIDE score in two groups. *p < 0.05, **p < 0.01, ***p < 0.001.

The GSVA results displayed in Figure 6A, the high-risk group genes are enriched in ABC transporters, apoptosis, RIG-I-like receptor signaling pathway, cell-cytokine receptor interaction, glucose metabolism, pyrimidine metabolism, and sulfur metabolism pathways. These pathways are closely associated with tumor development, and the ABC transporters were closely related to the multidrug resistance (MDR) of the tumor.

### Tumor microenvironment and immune-correlation analysis of the model

For confirming if the risk model was associated with tumor microenvironment and tumor immunity, we analyzed the differences in TME scoring, immune cells, and immune checkpoint gene expression between the two groups. As shown in Figure 6B, the results of the ESTIMATE analysis showed that the TME-related scores of the high-risk group were higher than those of the low-risk group. Moreover, we used the ssGSEA method for evaluating differences in immune cell infiltration between the two groups. The heatmap of immune cell expression is shown in Figure 6C, and the different analysis results are shown in Figure 6D. As Figure 6D indicates, the expression of T cells CD8, T cells CD4 memory resting, T cells CD4 memory activated, T cells follicular helper, NK cells resting, Monocytes, Macrophages M1, Macrophages M2 and Mast cells resting had significant differences between the two groups. Among them, within the high-risk group, T cells CD8, T cells CD4 memory activated, T cells follicular helper, and Macrophages M1 proportion were significantly increased, while the opposite results occurred in T cells CD4 memory resting, NK cells resting, Monocytes, Macrophages M2, and Mast cells resting proportions. Additionally, the results of correlation analysis demonstrated that the risk score and the expression of SMARCD3 were positively correlated with the expression of many immune checkpoints (Figure 6E), and as Figure 6F indicates, significant differences were found in immune checkpoint expression. Many immune checkpoints were upregulated in high-risk subgroups, such as PDCD1/PD-L1, BLTA, CTLA4, LAG3, CD276, TNFRSF25, IDO1, SIGLEC7, TIGIT, LILRB2, and so on. Moreover, the TIDE score of the high-risk group was significantly lower than that of the low-risk group (*p* < 0.01) (Figure 6G), which suggested that patients in the high-risk group may have a better response to immune checkpoint blockade (ICB) treatment.

### Analysis of signature genes in the risk model

**FIGURE 7:**
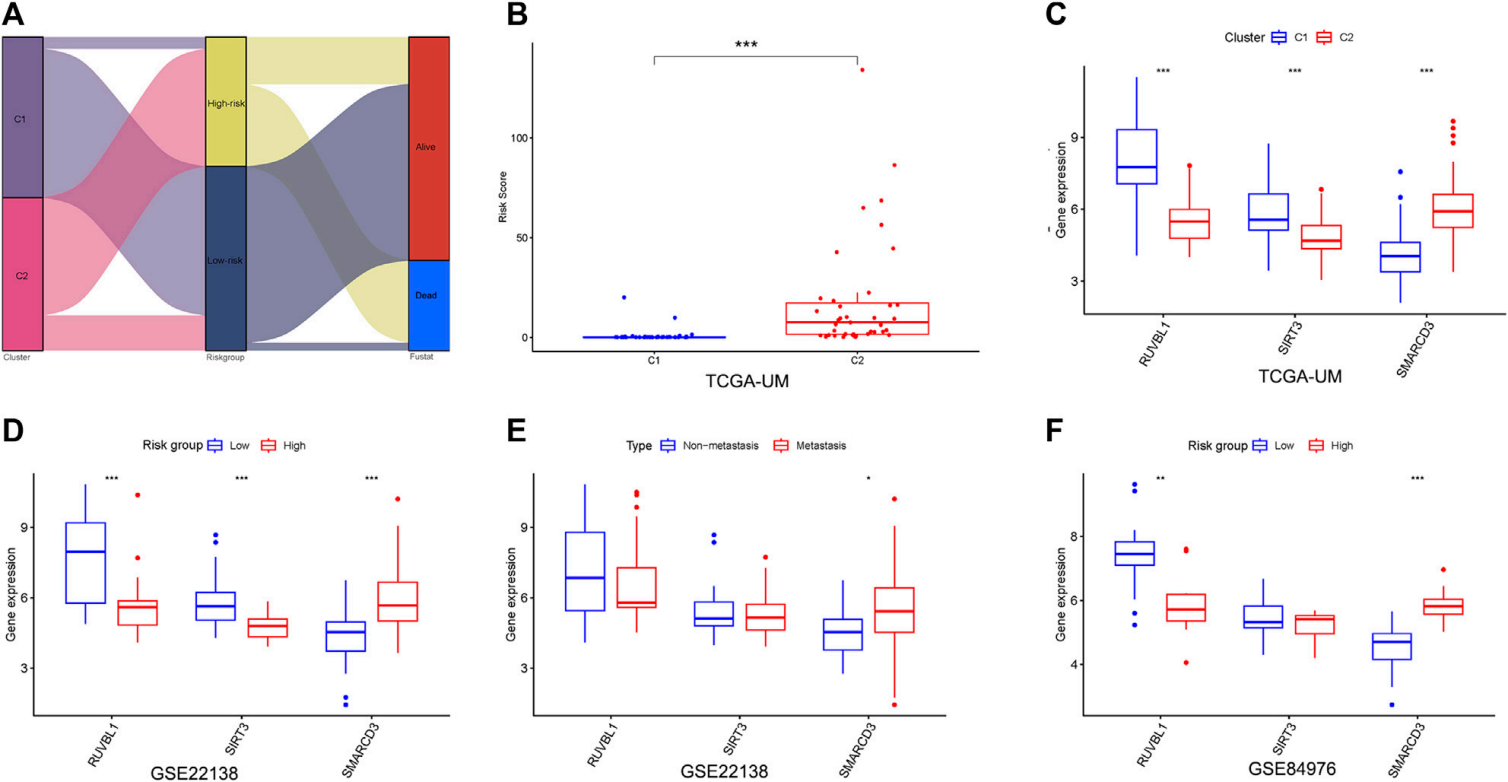
Analysis of signature genes in the risk model. **(A)** Sankey diagram of the two subgroups, two risk groups, and two clinical outcomes. **(B)** Differences in the risk scores between the two subgroups. **(C)** Differential expression of three signature genes in subtypes. **(D,E)** Differential expression of three signature genes in risk groupings for datasets GSE22138 and GSE84976. **(F)** Differential expression of three signature genes in the metastasis subgroup of GSE22138. *p < 0.05, **p < 0.01, ***p < 0.001.

**FIGURE 8:**
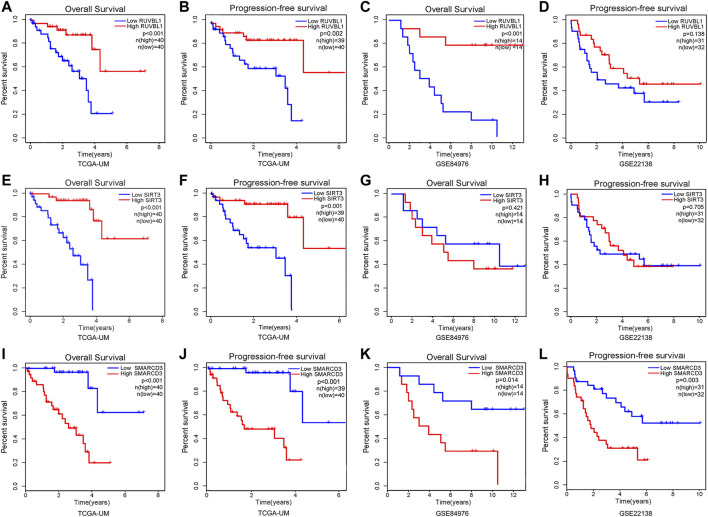
Survival analysis of signature genes in the risk model. Red represents patients in the high gene expression group and blue represents patients in the low-risk group. The x-axis represents the survival time and the y-axis represents the survival rate. **(A–D)** Survival analysis of OS and PFS in the RUVBL1 expression subgroup. **(E–H)** Survival analysis of OS and PFS in the SIRT3 expression subgroup. **(I–L)** Survival analysis of OS and PFS in the SMARCD3 expression subgroup.

First, we found the distribution and relationship of two subgroups, two risk groups, and two clinical outcomes (Figure 7A). And statistical and consistency analysis was performed on 80 UM patients in TCGA for subtypes and risk groupings, and the results were presented as a 2 × 2 contingency table (Supplementary Table S3), and the result of the consistency test Kappa coefficient (κ = 0.691) showed a substantial consistency in the classification of UM samples by tumor subtypes and risk groupings. We found a significant difference between the two subgroups in risk score (Figure 7B). Additionally, the relative expression of the signature gene SMARCD3 was higher in the poor prognosis group (C2 and high-risk group), and RUVBL1 expression in the better prognosis group (C1 and low-risk group) in the three datasets, but the SIRT3 expression was higher in the TCGA subtype and the group with good prognosis in the dataset GSE22138, while no difference was found in the risk group of the dataset GSE84976 (Figures 7C–E). Interestingly, the difference analysis results of the three signature genes between the group’s metastasis and non-metastasis in data set GSE22138 are shown in Figure 7F, the SMARCD3 expression is different, and the expression is higher in the group with metastasis. Moreover, the lower RUVBL1 and SIRT3 expressions were significantly correlated with poor survival probability (Figures 8A–C, E–G), and the higher expression of SMARCD3 was significantly correlated with poor survival probability (Figures 8I–L). Notably, RUVBL1 and SIRT3 expressions were not associated with progression-free survival in dataset 22138 (Figures 8D, H). These results suggest that the SMARCD3 gene may be closely associated with the progression of UM.

## Discussion

UM is the most common primary malignant intraocular tumor in adults and is characterized by high mortality (>95%), high metastasis (>50%), and poor prognosis within 5 years, making the search for effective biomarkers for accurate diagnosis, assessing prognosis and guiding treatment crucial [44]. CRs play various roles in tumorigenesis. It is a diagnostic and prognostic marker for many cancers [45–50].

In this study, we synthesized and analyzed the UM dataset collected from TCGA and GEO and developed a risk model for UM prognosis consisting of 3 CRs. Unlike other tumors, the UM dataset lacks normal tissue, and we used the NMF method to classify UM patients into two subtypes. The results of the survival analysis showed significant differences in overall survival and progression-free survival between the two subtypes. The results confirm that the expression of prognosis-related CRs based on the prognosis helps to identify UM subtypes. We obtained a risk model by the LASSO Cox regression model before which we improved the generalization ability of the model by preventing “overfitting” by randomly grouping the TCGA dataset. The survival analysis of the model was statistically significant in both the training and validation cohorts. The results of ROC curves comparing risk scores with other clinical traits for prognosis prediction of UM also confirmed the better prediction accuracy of the risk model, while the nomogram constructed based on clinical characteristics and risk scores could systematically predict OS of UM patients.

We further analyzed the TME and immune cell infiltration differences in subtypes and risk subgroups. Immune cells and stromal cells are the two major non-tumor components of the TME, and the ratio of these two cells has an important impact on tumor prognosis. ESTIMATE (Estimation of STromal and Immune cells in MAlignant Tumour tissues using Expression data) was calculated by analyzing transcriptional data from cancer samples to calculate the proportion of relevant immune, stromal, and tumor cells in the TME [51]. In our study, stromal score, immune score, and ESTIMATE score were significantly higher in the poor prognosis groups than in the better prognosis groups, suggesting that the prognosis of UM was associated with non-tumor cell infiltration. It has been demonstrated that the poor prognosis of UM is positively correlated with immune cell infiltration [52], so we further analyzed the differences in immune cell infiltration. The infiltration of T-cell CD8, T-cell CD4 memory activation, T-cell follicular helper cells, and macrophages was higher in the poorer prognosis group (C2 and high-risk group) than in the better prognosis group (C1 and low-risk group). These types of immune cells were also closely associated with tumor development. For example, CD8^+^ T cells themselves can selectively detect and eradicate cancer cells, but tumors continue to develop when they coexist with tumor cells, which is associated with dysfunctional tumor-responsive CD8^+^ T cells [53]. Accumulation of T follicular helper (Tfh) cells has positive or negative prognostic effects in different human cancers. In melanoma, Tfh cells exert an immunosuppressive function and suppress the function of CD8^+^ T cells. Additional studies have shown that high Tfh levels are associated with an increase in CD8^+^ T cells and that CD8+/Tfh crosstalk plays an important role in shaping the antitumor immune response generated by immunotherapy [54–56]. Infiltration of CD4 memory-activated T cells may be a poor prognostic factor in many cancers [57–60]. In cutaneous melanoma, high infiltration of CD4 memory-activated T cells promotes melanoma metastasis [61]. Macrophages play an important role in tumorigenesis and metastasis [62, 63]. In uveal melanoma, macrophages are a negative prognostic factor for it [64]. The mechanisms by which they influence the progression and prognosis of UM need further investigation. In addition, we performed a TIDE analysis between the high- and low-risk groups. Tumor immune dysfunction and rejection (TIDE) is a computational framework for identifying factors underlying both mechanisms of tumor immune escape, which include, in some tumors, high levels of cytotoxic T-cell infiltration but dysfunction of these T cells, and in other tumors, immunosuppressive factors that may preclude T-cell infiltration of the tumor [65–67]. The TIDE score can predict the immune checkpoint suppression efficacy, and the results of the TIDE score will better help physicians to select patients more suitable for immune checkpoint blockade (ICB) therapy [68]. Our study showed that the TIDE score was significantly lower in the high-risk group than in the low-risk group, which also suggests that patients in the high-risk group may be more effective when receiving ICB therapy. Many immune checkpoint genes expression was higher in the high-risk group with a poorer prognosis, and we showed significant correlations between risk model scores and three signature genes and many immune checkpoint genes expression, which also suggests that patients in the high-risk group may be more effective when treated with immune checkpoint blockade.

Our risk model consisted of RUVBL1, SIRT3, and SMARCD3. These three genes have also been reported several times in previous studies to play a role in the development of tumors. Sirtuin 3(SIRT3)is the most talked about Sirtuin family member in recent times (a family of NAD+-dependent deacetylases that regulate signaling pathways involved in cellular proliferation and differentiation, metabolism, response to stress, and cancer. Recent studies have pointed out that SIRT3 is a critical regulator of cell metabolism and played a dual role in cancer, as it can act as a suppressor or promoter in a variety of tumors [69], such as breast cancer, colon cancer, and prostate cancer [70–76]. Our findings suggest that SIRT3 may act as a protective factor in the development of uveal melanoma, which is similar to previous findings [77]. However, the specific role and molecular mechanisms of SIRT3 in UM have not been reported. SMARCD3 (SWI-SNF-associated, matrix-associated, actin-dependent regulator of chromatin, subfamily d, member 3 protein) is an important member of the SWI/SNF chromatin remodeling complex, has a role in regulating gene expression [78, 79]. Previous studies have confirmed that SMARCD3 has a cancer-promoting effect in ER+ breast cancer [80, 81]. The results of the related bioinformatic analysis also suggested that SMARCD3 was a prognostic and potential treatment target maker for colorectal cancer, neuroblastoma, hematologic malignancy, and uveal melanoma [82–85]. RUVBL1 belongs to the AAA+ superfamily of ATPases and plays an important role in many cellular activities [86]. It has been widely reported as an oncogenic factor. For example, it can accelerate the progression of lung cancer by activating the RAF/MEK/ERK pathway, and the high expression of RUVBL1 in mammary carcinoma suggests a worse prognosis [87, 88]. However, a recent study found that a decrease in RUVBL1 promoted the progression of hepatocellular carcinoma [89]. In our study, SMARCD3 expression was negatively correlated with prognosis. OS and PFS were significantly different between high and low expression groups, while SMARCD3 expression levels differed both between subtypes and between risk subgroups, with higher SMARCD3 expression levels in the poorer prognosis group and higher SMARCD3 expression levels in the metastatic group in dataset GSE22138. The expression of SIRT3 and RUVBL1 was positively correlated with prognosis, with differential and survival analyses showing opposite trends to SMARCD3. These results also suggest that SMARCD3 may play a facilitating role in UM progression, while SIRT3 and RUVBL1 play a protective role. This of course requires further experiments to verify.

Notably, we pioneered the construction of a risk model consisting of three CRs genes that can predict the prognosis of UM. Our risk model consists of only three genes, which reduces the cost of clinical testing and improves the possibility of clinical application. Our research offers new insight into the projection of UM but still has some limitations. First, the sample data in this study were from predominantly white Western countries, and there may be genetic inheritance differences. Second, the lack of normal or paracancerous tissues compared with tumor tissues made it impossible to observe the expression of the three characteristic genes in normal and tumor tissues, which needs to be demonstrated with more samples. In addition, all three genes included in our risk model are involved in tumor biological processes. However, the mechanism of how these CRs regulate the biological behavior of UM cells needs to be validated experimentally.

## Conclusion

In conclusion, we successfully constructed a risk model consisting of only three signature chromatin regulators that are valuable in predicting the prognosis of uveal melanoma patients. In addition, the model interacts closely with the tumor immune environment and TIDE score results, which may facilitate the development of new therapies for uveal melanoma treatment.

## Data Availability

The data in this study are publicly available, and readers can request them by contacting the corresponding author’s email address.
