# Supplementary figures and images for: Tumor subtypes and signature model construction based on chromatin regulators for better prediction of prognosis in uveal melanoma
Source: Pathol Oncol Res. 2023 Jun 9;29:1610980. doi: 10.3389/pore.2023.1610980 (PMC10287976; doi:10.3389/pore.2023.1610980)

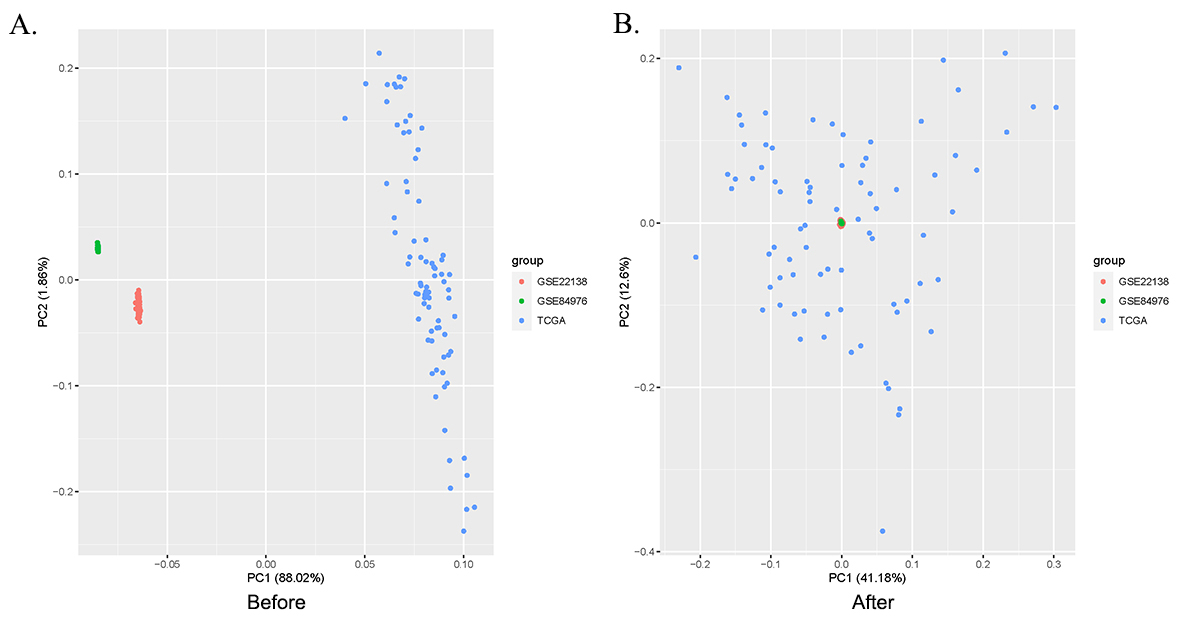

Supplement: Supplementary file 1 [file Image1.JPEG]
