# Supplementary material for: Tumor subtypes and signature model construction based on chromatin regulators for better prediction of prognosis in uveal melanoma
Source: Pathol Oncol Res. 2023 Jun 9;29:1610980. doi: 10.3389/pore.2023.1610980 (PMC10287976; doi:10.3389/pore.2023.1610980)
Supplement: Supplementary file 2 [file DataSheet1.docx]

**Table S1 Genelist of Chromatin Regulators**

| **Genename** | **Genename** | **Genename** | **Genename** | **Genename** |
| --- | --- | --- | --- | --- |
| A1CF | DDB1 | KAT6A | PHF7 | SRCAP |
| ACTB | DDB2 | KAT6B | PHF8 | SRRM2 |
| ACTL6A | DDX21 | KAT7 | PHIP | SRSF1 |
| ACTL6B | DDX50 | KAT8 | PIWIL4 | SRSF3 |
| ACTR2 | DEK | KDM1A | PKM | SS18L1 |
| ACTR3 | DHX30 | KDM1B | PKN1 | SS18L2 |
| ACTR3B | DIDO1 | KDM2A | POGZ | SSRP1 |
| ACTR5 | DMAP1 | KDM2B | POLE3 | STK31 |
| ACTR6 | DNAJC1 | KDM3A | PPARGC1A | STK4 |
| ACTR8 | DNAJC2 | KDM3B | PPM1G | SUDS3 |
| ADNP | DND1 | KDM4A | PPP2CA | SUPT16H |
| AEBP2 | DNMT1 | KDM4B | PPP4C | SUPT3H |
| AHCTF1 | DNMT3A | KDM4C | PPP4R2 | SUPT6H |
| AICDA | DNMT3B | KDM4D | PPP4R3A | SUPT7L |
| AIRE | DNMT3L | KDM4E | PPP4R3B | SUV39H1 |
| AKAP1 | DNTT | KDM5A | PPP4R3CP | SUV39H2 |
| ALKBH1 | DNTTIP2 | KDM5B | PRC1 | SUZ12 |
| ALKBH3 | DOT1L | KDM5C | PRDM1 | SYNCRIP |
| ANP32A | DPF1 | KDM5D | PRDM10 | TADA1 |
| ANP32B | DPF2 | KDM6A | PRDM11 | TADA2A |
| ANP32E | DPF3 | KDM6B | PRDM12 | TADA2B |
| APBB1 | DPPA3 | KDM7A | PRDM13 | TADA3 |
| APEX1 | DPY30 | KDM8 | PRDM14 | TAF1 |
| APOBEC1 | DR1 | KEAP1 | PRDM15 | TAF10 |
| APOBEC2 | DTX3L | KMT2A | PRDM16 | TAF12 |
| APOBEC3A | DUSP1 | KMT2B | PRDM2 | TAF1L |
| APOBEC3B | DZIP3 | KMT2C | PRDM4 | TAF2 |
| APOBEC3C | E2F6 | KMT2D | PRDM5 | TAF3 |
| APOBEC3D | EED | KMT2E | PRDM6 | TAF4 |
| APOBEC3F | EHMT1 | KMT5A | PRDM7 | TAF5 |
| APOBEC3G | EHMT2 | KMT5B | PRDM8 | TAF5L |
| APOBEC3H | EID1 | KMT5C | PRDM9 | TAF6 |
| ARID1A | EID2 | L3MBTL1 | PRKAA1 | TAF6L |
| ARID1B | EID2B | L3MBTL2 | PRKAA2 | TAF7 |
| ARID2 | ELP2 | L3MBTL3 | PRKAB1 | TAF8 |
| ARID3A | ELP3 | L3MBTL4 | PRKAB2 | TAF9 |
| ARID3B | ELP4 | LAS1L | PRKAG1 | TAF9B |
| ARID3C | ELP5 | LBR | PRKAG2 | TBL1XR1 |
| ARID4A | ELP6 | LEO1 | PRKAG3 | TCF4 |
| ARID4B | EMSY | LMNA | PRKCA | TDG |
| ARID5A | ENY2 | LMNB1 | PRKCB | TDRD1 |
| ARID5B | EP300 | LMNB2 | PRKCD | TDRD12 |
| ARNTL | EP400 | LRWD1 | PRKDC | TDRD3 |
| ARRB1 | EPC1 | MAP3K7 | PRMT1 | TDRD5 |
| ASCL1 | EPC2 | MAPKAPK3 | PRMT2 | TDRD7 |
| ASCL2 | ERBB4 | MASTL | PRMT3 | TDRD9 |
| ASF1A | ERCC6 | MAX | PRMT5 | TDRKH |
| ASF1B | ERCC6L | MAZ | PRMT6 | TERF1 |
| ASH1L | ERCC6L2 | MBD1 | PRMT7 | TERF2 |
| ASH2L | EXOSC1 | MBD2 | PRMT8 | TET1 |
| ASXL1 | EXOSC2 | MBD3 | PRMT9 | TET2 |
| ASXL2 | EXOSC3 | MBD4 | PRPF31 | TET3 |
| ASXL3 | EXOSC4 | MBD5 | PRR12 | TEX10 |
| ATAD2 | EXOSC5 | MBD6 | PRR14 | TFDP1 |
| ATAD2B | EXOSC6 | MBIP | PSIP1 | TFF1 |
| ATF2 | EXOSC7 | MBTD1 | PWWP2B | TFPT |
| ATF7IP | EXOSC8 | MCRS1 | PYGO1 | TLE1 |
| ATM | EXOSC9 | MDC1 | PYGO2 | TLE2 |
| ATN1 | EYA1 | MDM2 | RAC3 | TLE4 |
| ATR | EYA2 | MDM4 | RAD51 | TLK1 |
| ATRX | EYA3 | MEAF6 | RAD54B | TLK2 |
| ATXN7 | EYA4 | MECOM | RAD54L | TNP1 |
| ATXN7L3 | EZH1 | MECP2 | RAD54L2 | TNP2 |
| AURKA | EZH2 | MEN1 | RAG1 | TONSL |
| AURKB | FAM175A | MGA | RAG2 | TOP2A |
| AURKC | FAM175B | MGEA5 | RAI1 | TOP2B |
| BABAM1 | FBL | MGMT | RARA | TOPBP1 |
| BAG6 | FBRS | MIER1 | RB1 | TOX |
| BAHD1 | FBRSL1 | MIER2 | RBBP4 | TOX2 |
| BANF1 | FBXL19 | MIER3 | RBBP5 | TOX3 |
| BANF2 | FMR1 | MINA | RBBP7 | TOX4 |
| BANP | FOXA1 | MIS18BP1 | RBP1 | TP53 |
| BAP1 | FOXO1 | MLLT1 | RBX1 | TP53BP1 |
| BARD1 | FOXP1 | MLLT10 | RCC1 | TRDMT1 |
| BAZ1A | FOXP2 | MLLT3 | RCOR1 | TRIM16 |
| BAZ1B | FOXP3 | MLLT6 | RCOR2 | TRIM24 |
| BAZ2A | FOXP4 | MOCS1 | RCOR3 | TRIM27 |
| BAZ2B | FTO | MORF4 | REST | TRIM28 |
| BBX | FXR1 | MORF4L1 | REV1 | TRIM33 |
| BCL10 | FXR2 | MORF4L2 | RING1 | TRRAP |
| BCOR | GABRG1 | MOV10 | RIT1 | TSPY1 |
| BCORL1 | GADD45A | MPHOSPH8 | RLIM | TSPYL1 |
| BMI1 | GADD45B | MRGBP | RMI1 | TSPYL2 |
| BOD1 | GADD45G | MSH6 | RNF168 | TSPYL4 |
| BPTF | GATAD1 | MSL1 | RNF17 | TSPYL5 |
| BRCA1 | GATAD2A | MSL2 | RNF2 | TSPYL6 |
| BRCA2 | GATAD2B | MSL3 | RNF20 | TSSK6 |
| BRCC3 | GFI1 | MST1 | RNF40 | TTF2 |
| BRD1 | GFI1B | MTA1 | RNF8 | TTK |
| BRD2 | GLYATL1 | MTA2 | RPS6KA3 | TXN2 |
| BRD3 | GLYR1 | MTA3 | RPS6KA4 | TYW5 |
| BRD4 | GSE1 | MTF2 | RPS6KA5 | UBE2A |
| BRD7 | GSG2 | MUM1 | RRP8 | UBE2B |
| BRD8 | GTF2I | MYBBP1A | RSAD1 | UBE2D1 |
| BRD9 | GTF3C1 | MYO1C | RSF1 | UBE2D3 |
| BRDT | GTF3C4 | MYSM1 | RTF1 | UBE2E1 |
| BRE | HAT1 | NAA60 | RUVBL1 | UBE2H |
| BRMS1 | HBP1 | NAP1L1 | RUVBL2 | UBE2N |
| BRMS1L | HCFC1 | NAP1L2 | RYBP | UBE2T |
| BRPF1 | HCFC2 | NAP1L3 | SAFB | UBN1 |
| BRPF3 | HDAC1 | NAP1L4 | SAP130 | UBR2 |
| BRWD1 | HDAC10 | NAP1L5 | SAP18 | UBR5 |
| BRWD3 | HDAC11 | NASP | SAP25 | UBR7 |
| BTAF1 | HDAC2 | NAT10 | SAP30 | UBTF |
| BUB1 | HDAC3 | NBN | SAP30L | UCHL5 |
| C14orf169 | HDAC4 | NCL | SATB1 | UHRF1 |
| C17orf49 | HDAC5 | NCOA1 | SATB2 | UHRF2 |
| CARM1 | HDAC6 | NCOA2 | SCMH1 | UIMC1 |
| CBX1 | HDAC7 | NCOA3 | SCML1 | UNK |
| CBX2 | HDAC8 | NCOA4 | SCML2 | USP11 |
| CBX3 | HDAC9 | NCOA5 | SCML4 | USP12 |
| CBX4 | HDGF | NCOA6 | SENP1 | USP15 |
| CBX5 | HELLS | NCOA7 | SENP3 | USP16 |
| CBX6 | HIF1AN | NCOR1 | SET | USP17L2 |
| CBX7 | HINFP | NCOR2 | SETBP1 | USP21 |
| CBX8 | HIRA | NEK6 | SETD1A | USP22 |
| CDC6 | HIRIP3 | NEK9 | SETD1B | USP3 |
| CDC73 | HJURP | NFRKB | SETD2 | USP36 |
| CDK1 | HLCS | NFYB | SETD3 | USP44 |
| CDK17 | HLTF | NFYC | SETD4 | USP46 |
| CDK2 | HMG20A | NIPBL | SETD5 | USP49 |
| CDK3 | HMG20B | NOC2L | SETD6 | USP7 |
| CDK5 | HMGA1 | NPAS2 | SETD7 | UTY |
| CDK7 | HMGA2 | NPM1 | SETD8P1 | VDR |
| CDK9 | HMGB1 | NPM2 | SETD9 | VEZF1 |
| CDY1 | HMGB2 | NSD1 | SETDB1 | VPS72 |
| CDY2A | HMGB3 | NSL1 | SETDB2 | VRK1 |
| CDYL | HMGN1 | OGT | SETMAR | WAC |
| CDYL2 | HMGN2 | ORC1 | SF3B1 | WDR5 |
| CECR2 | HMGN3 | ORC2 | SF3B3 | WDR77 |
| CENPC | HMGN4 | PADI1 | SFMBT1 | WDR82 |
| CHAF1A | HMGN5 | PADI2 | SFMBT2 | WHSC1 |
| CHAF1B | HN1 | PADI3 | SFPQ | WHSC1L1 |
| CHD1 | HN1L | PADI4 | SGF29 | WSB2 |
| CHD1L | HNF1A | PAF1 | SHPRH | XRCC1 |
| CHD2 | HNRNPA1 | PAGR1 | SIN3A | YAF2 |
| CHD3 | HP1BP3 | PAK2 | SIN3B | YEATS2 |
| CHD4 | HR | PARG | SIRT1 | YEATS4 |
| CHD5 | HSPA1A | PARP1 | SIRT2 | YWHAB |
| CHD6 | HUWE1 | PARP2 | SIRT3 | YWHAE |
| CHD7 | IDH1 | PARP3 | SIRT4 | YWHAZ |
| CHD8 | IDH2 | PARP4 | SIRT5 | YY1 |
| CHD9 | IFIT3 | PATZ1 | SIRT6 | ZBTB16 |
| CHEK1 | IGFBP7 | PAXIP1 | SIRT7 | ZBTB24 |
| CHMP1A | IKBKAP | PBK | SKP1 | ZBTB33 |
| CHMP1B | IKZF1 | PBRM1 | SLF1 | ZBTB38 |
| CHRAC1 | IKZF3 | PCGF1 | SMARCA1 | ZBTB4 |
| CHTOP | ING1 | PCGF2 | SMARCA2 | ZBTB7C |
| CHUK | ING2 | PCGF3 | SMARCA4 | ZCWPW1 |
| CIC | ING3 | PCGF5 | SMARCA5 | ZCWPW2 |
| CIR1 | ING4 | PCGF6 | SMARCAD1 | ZFAT |
| CIT | ING5 | PCNA | SMARCAL1 | ZFP57 |
| CLNS1A | INO80 | PDP1 | SMARCB1 | ZGPAT |
| CLOCK | INO80B | PDS5A | SMARCC1 | ZHX1 |
| CRB2 | INO80C | PDS5B | SMARCC2 | ZHX2 |
| CREBBP | INO80D | PELP1 | SMARCD1 | ZHX3 |
| CSNK2A1 | INO80E | PES1 | SMARCD2 | ZMYM1 |
| CSRP2BP | JADE1 | PHC1 | SMARCD3 | ZMYM2 |
| CTBP1 | JADE2 | PHC2 | SMARCE1 | ZMYM3 |
| CTBP2 | JADE3 | PHC3 | SMC1A | ZMYM4 |
| CTCF | JAK2 | PHF1 | SMCHD1 | ZMYM5 |
| CTCFL | JARID2 | PHF10 | SMYD1 | ZMYM6 |
| CTR9 | JDP2 | PHF12 | SMYD2 | ZMYND11 |
| CUL1 | JMJD1C | PHF13 | SMYD3 | ZMYND8 |
| CUL2 | JMJD4 | PHF14 | SMYD4 | ZNF217 |
| CUL3 | JMJD6 | PHF19 | SMYD5 | ZNF516 |
| CUL4A | JMJD7 | PHF2 | SNAI2 | ZNF532 |
| CUL4B | JMJD8 | PHF20 | SND1 | ZNF541 |
| CUL5 | KANSL1 | PHF20L1 | SP1 | ZNF592 |
| CXXC1 | KANSL2 | PHF21A | SP100 | ZNF687 |
| DAPK3 | KANSL3 | PHF21B | SP110 | ZNF711 |
| DAXX | KAT2A | PHF23 | SP140 | ZNHIT1 |
| DBF4 | KAT2B | PHF3 | SPEN | ZRANB3 |
| DBF4B | KAT5 | PHF6 | SPOP | ZZZ3 |

**Table S2 Results of univariate analysis of genes associated with UM prognosis**

| **Genename** | **HR** | **HR.95L** | **HR.95H** | **Pvalue** |
| --- | --- | --- | --- | --- |
| ACTB | 1.0041405 | 1.002102 | 1.0061832 | 6.74E-05 |
| ACTR3 | 1.1337454 | 1.0584849 | 1.214357 | 0.0003412 |
| ANP32E | 1.0922562 | 1.0235287 | 1.1655985 | 0.0077832 |
| APBB1 | 0.8566948 | 0.7654837 | 0.9587742 | 0.0070825 |
| APOBEC3D | 286.42968 | 7.9555442 | 10312.552 | 0.0019734 |
| APOBEC3G | 1.1714548 | 1.042146 | 1.3168082 | 0.0080082 |
| ARID5A | 1.0583918 | 1.0243373 | 1.0935784 | 0.0006714 |
| ATAD2 | 1.4609917 | 1.1979545 | 1.7817845 | 0.0001816 |
| AURKA | 1.4754928 | 1.1884026 | 1.8319372 | 0.000426 |
| BAP1 | 0.9359695 | 0.9048007 | 0.9682121 | 0.0001285 |
| BAZ1A | 1.3938096 | 1.106378 | 1.7559146 | 0.0048341 |
| BAZ1B | 1.1842372 | 1.0429484 | 1.3446664 | 0.0090887 |
| BRD3 | 0.6006513 | 0.4402289 | 0.8195327 | 0.0013028 |
| BRPF1 | 0.3333156 | 0.191644 | 0.579717 | 9.99E-05 |
| BUB1 | 2.179977 | 1.3702255 | 3.4682612 | 0.0010038 |
| CBX3 | 1.0427819 | 1.0139491 | 1.0724346 | 0.0034085 |
| CDC6 | 2.9626837 | 1.5624118 | 5.617914 | 0.0008785 |
| CDK2 | 0.9731519 | 0.958666 | 0.9878567 | 0.0003756 |
| CDK5 | 1.1225429 | 1.0424938 | 1.2087386 | 0.0021951 |
| CDYL2 | 4.2578913 | 1.6680752 | 10.868598 | 0.0024445 |
| CHD7 | 1.4201016 | 1.1876193 | 1.6980935 | 0.0001205 |
| CHRAC1 | 1.1162364 | 1.0602033 | 1.1752308 | 2.85E-05 |
| CTBP1 | 1.2405219 | 1.0736515 | 1.4333278 | 0.0034545 |
| CTCFL | 2.65E+165 | 1.08E+64 | 6.48E+266 | 0.0013847 |
| CUL1 | 1.106876 | 1.0286087 | 1.1910986 | 0.0066509 |
| CXXC1 | 0.7348103 | 0.5873676 | 0.9192645 | 0.0070038 |
| DAXX | 0.8658958 | 0.7913644 | 0.9474465 | 0.0017154 |
| DHX30 | 0.7739153 | 0.678463 | 0.8827967 | 0.0001356 |
| EED | 1.8131101 | 1.1989829 | 2.7417975 | 0.0048025 |
| EHMT2 | 0.8701418 | 0.7844203 | 0.965231 | 0.0085704 |
| ENY2 | 1.1294139 | 1.0571948 | 1.2065665 | 0.0003066 |
| EXOSC4 | 1.0189771 | 1.0094498 | 1.0285943 | 8.77E-05 |
| EXOSC8 | 1.2624784 | 1.0581228 | 1.5063012 | 0.0096802 |
| EYA4 | 115.92657 | 7.9026324 | 1700.5689 | 0.0005233 |
| EZH2 | 2.4142542 | 1.4748904 | 3.9519028 | 0.0004559 |
| FOXP4 | 0.903478 | 0.8408691 | 0.9707485 | 0.0056023 |
| FXR1 | 0.8804894 | 0.7997312 | 0.9694027 | 0.0095123 |
| HDAC10 | 774.08687 | 18.373952 | 32611.954 | 0.0004919 |
| HDAC11 | 0.7915112 | 0.6974275 | 0.898287 | 0.0002931 |
| HDAC4 | 1.1841352 | 1.0979198 | 1.2771208 | 1.18E-05 |
| HDAC6 | 0.7507213 | 0.626105 | 0.9001405 | 0.0019619 |
| HDAC8 | 19.307385 | 2.2025456 | 169.2474 | 0.0075207 |
| HIF1AN | 0.56151 | 0.364244 | 0.8656106 | 0.0089615 |
| HMGA2 | 2.911E+14 | 10401.371 | 8.15E+24 | 0.0066554 |
| HMGB3 | 1.287037 | 1.0813855 | 1.531798 | 0.0045 |
| IDH2 | 1.0708327 | 1.0441669 | 1.0981795 | 1.043E-07 |
| IGFBP7 | 1.0080796 | 1.0040493 | 1.0121262 | 8.25E-05 |
| IKZF3 | 1.5968458 | 1.1503318 | 2.216679 | 0.0051598 |
| JARID2 | 0.7136571 | 0.5920253 | 0.8602781 | 0.0004022 |
| LEO1 | 1.2522354 | 1.0746762 | 1.4591312 | 0.003938 |
| MAPKAPK3 | 0.7968002 | 0.6978662 | 0.9097597 | 0.0007847 |
| MASTL | 2.3359038 | 1.2360303 | 4.4144925 | 0.0089887 |
| MBD2 | 1.3018849 | 1.1090434 | 1.5282579 | 0.0012584 |
| MBD5 | 6.6244913 | 1.6454457 | 26.669908 | 0.0077958 |
| MDC1 | 0.7420241 | 0.5982379 | 0.9203693 | 0.0066268 |
| MLLT1 | 0.8809837 | 0.8133057 | 0.9542934 | 0.0018892 |
| NASP | 1.2335204 | 1.0679237 | 1.4247952 | 0.0043247 |
| NBN | 1.0822896 | 1.029714 | 1.1375497 | 0.0018556 |
| NCOA2 | 1.1738954 | 1.0558832 | 1.3050973 | 0.0030181 |
| NEK9 | 1.1500631 | 1.035189 | 1.2776847 | 0.0092118 |
| PADI1 | 2.4924734 | 1.6678592 | 3.7247891 | 8.36E-06 |
| PAF1 | 1.0795923 | 1.0217414 | 1.1407188 | 0.0064226 |
| PARP2 | 1.4933136 | 1.1232288 | 1.9853351 | 0.0057851 |
| PARP3 | 0.7614874 | 0.6260164 | 0.9262745 | 0.0064068 |
| PARP4 | 1.1616642 | 1.0602125 | 1.2728239 | 0.0013091 |
| PCNA | 1.0276949 | 1.010451 | 1.045233 | 0.0015552 |
| PHC2 | 0.9438674 | 0.9079494 | 0.9812063 | 0.0035181 |
| PHF1 | 0.8711992 | 0.8043196 | 0.9436399 | 0.0007158 |
| PHF20L1 | 1.4590535 | 1.1249997 | 1.8923002 | 0.0044018 |
| PRDM1 | 3.8087686 | 1.7343422 | 8.3643921 | 0.0008628 |
| PRDM11 | 0.3076396 | 0.145313 | 0.6512987 | 0.0020669 |
| PRDM12 | 47859805 | 1691.712 | 1.354E+12 | 0.0007214 |
| PRKCD | 0.8627474 | 0.7973396 | 0.9335208 | 0.0002424 |
| PRKDC | 1.0458728 | 1.0218647 | 1.0704449 | 0.0001534 |
| PRPF31 | 1.2314258 | 1.0849813 | 1.3976364 | 0.0012704 |
| RAD54B | 19.028906 | 3.5018459 | 103.4024 | 0.0006469 |
| RBX1 | 1.1880424 | 1.092787 | 1.2916009 | 5.33E-05 |
| RCOR2 | 1.1879639 | 1.0636932 | 1.3267531 | 0.0022488 |
| RING1 | 0.9665172 | 0.9432806 | 0.9903263 | 0.0060903 |
| RUVBL1 | 0.674241 | 0.53713 | 0.8463518 | 0.0006785 |
| SAP18 | 1.1289013 | 1.0614822 | 1.2006025 | 0.0001138 |
| SAP30 | 1.3920082 | 1.151763 | 1.6823659 | 0.0006223 |
| SATB1 | 0.3383508 | 0.1703773 | 0.6719279 | 0.0019625 |
| SATB2 | 3.8570453 | 1.5932435 | 9.3374292 | 0.002767 |
| SETBP1 | 34.568275 | 4.9468824 | 241.55934 | 0.0003547 |
| SF3B3 | 0.8912099 | 0.8219978 | 0.9662495 | 0.0052327 |
| SFMBT1 | 0.2172574 | 0.0869077 | 0.5431138 | 0.0010916 |
| SIRT3 | 0.317337 | 0.2074479 | 0.4854366 | 1.21E-07 |
| SMARCA5 | 1.0864844 | 1.0279053 | 1.1484018 | 0.0033542 |
| SMARCC1 | 0.8977948 | 0.8355266 | 0.9647037 | 0.0032843 |
| SMARCD3 | 1.5872 | 1.3190411 | 1.9098752 | 9.95E-07 |
| SMYD3 | 1.8082524 | 1.3356874 | 2.4480105 | 0.0001267 |
| STK4 | 1.3445719 | 1.0881812 | 1.6613719 | 0.006091 |
| TAF2 | 1.1561999 | 1.0637408 | 1.2566954 | 0.0006424 |
| TAF5 | 3.2788458 | 1.4914376 | 7.208367 | 0.0031314 |
| TDRD1 | 45.077492 | 2.8035822 | 724.77998 | 0.0072004 |
| TERF1 | 1.5771339 | 1.1986572 | 2.0751149 | 0.0011371 |
| TFDP1 | 1.1971532 | 1.0524244 | 1.3617849 | 0.0061964 |
| TFPT | 1.0861446 | 1.0368331 | 1.1378015 | 0.0004907 |
| TOP2A | 1.3099462 | 1.0795384 | 1.5895304 | 0.0062311 |
| TSPYL5 | 1.0646149 | 1.0230981 | 1.1078164 | 0.0020346 |
| UBE2E1 | 0.7222245 | 0.5700259 | 0.9150604 | 0.0070364 |
| UBE2T | 1.2484527 | 1.108528 | 1.4060394 | 0.0002534 |
| USP11 | 0.9549938 | 0.9230625 | 0.9880298 | 0.0079544 |
| YWHAB | 1.0544504 | 1.0170346 | 1.0932428 | 0.0040237 |
| YWHAZ | 1.0154159 | 1.0075969 | 1.0232955 | 0.0001049 |
| ZCWPW1 | 0.5216972 | 0.3241543 | 0.8396248 | 0.0073641 |
| ZHX3 | 1.5162473 | 1.1831182 | 1.9431752 | 0.0010074 |
| ZMYND8 | 1.1470777 | 1.0527083 | 1.2499068 | 0.0017324 |
| ZNF687 | 0.7450156 | 0.616559 | 0.9002356 | 0.0023006 |
| ZNHIT1 | 1.1078732 | 1.0479347 | 1.17124 | 0.0003064 |

**Table S3 The contingency table in Subtype and risk grouping**

|  | | Subtype | | Totel |
| --- | --- | --- | --- | --- |
|  |  | C1 | C2 |  |
| Risk group | Low | 38 | 9 | 47 |
|  | High | 3 | 30 | 33 |
| Totel | | 41 | 39 | 80 |
| κ=0.699 |  |  |  |  |
